# Novel Multiplex PCR Method and Genome Sequence-Based Analog for High-Resolution Subclonal Assignment and Characterization of Escherichia coli Sequence Type 131 Isolates

**DOI:** 10.1128/spectrum.01064-22

**Published:** 2022-05-23

**Authors:** Brian D. Johnston, David M. Gordon, Samantha Burn, Timothy J. Johnson, Bonnie P. Weber, Elizabeth A. Miller, James R. Johnson

**Affiliations:** a Minneapolis VA Health Care System, Minneapolis, Minnesota, USA; b University of Minnesota, Minneapolis, Minnesota, USA; c Research School of Biology, The Australian National University Australia, Canberra, Australian Capital Territory, Australia; d Department of Veterinary and Biomedical Sciences, College of Veterinary Medicine, University of Minnesota, Saint Paul, Minnesota, USA; Instituto de Higiene

**Keywords:** Escherichia coli, PCR, ST131, antimicrobial resistance, bioinformatics, clonality, diagnostics, genome sequencing, molecular subtyping

## Abstract

Escherichia coli sequence type 131 (ST131) is a pandemic, multidrug-resistant extraintestinal pathogen. The multiple distinctive ST131 subclones differ for *rfb* and *fliC* alleles (O and H antigens), *fimH* allele (type-1 fimbriae adhesin), resistance phenotype and genotype, clinical correlates, and host predilection. Current PCR assays for detecting ST131 and its main subclones offer limited sub-ST characterization. Here we combined 22 novel and 14 published primers for a multiplex PCR assay to detect and extensively characterize ST131 isolates. The primers target *mdh36*, *gyrB47, trpA72*, *sbmA, plsB, nupC, rmuC, kefC, ybbW*, the O16 and O25b *rfb* variants, five *fimH* alleles (*fimH22*, *fimH*27, *fimH*30, *fimH35*, and *fimH41*), two *fliC* alleles (H4 and H5), a (subclone-specific) fluoroquinolone resistance-associated *parC* allele, and a (subclone-specific) prophage marker. The resulting amplicons resolve 15 molecular subsets within ST131, including 3 within clade A (*H*41 subclone), 5 within clade B (*H*22 subclone), and 7 within clade C (*H*30 subclone), which includes subclones C0 (*H*30S: 2 subsets), C1 and C1-M27 (*H*30R1: 2 subsets), and C2 (*H*30Rx: 3 subsets). Validation in three laboratories showed that this assay provides a rapid, accurate, and portable method for rapidly detecting and characterizing E. coli ST131 and its key subsets. Additionally, for users with whole genome sequencing (WGS) capability, we developed a command-line executable called ST131Typer, an *in silico* version of the extended multiplex PCR assay. Its accuracy was 87.8%, with most issues due to incomplete or fragmented input genome assemblies. These two novel assays should facilitate detailed ST131 subtyping using either endpoint PCR or WGS.

**IMPORTANCE** These novel assays provide greater subclonal resolution and characterization of E. coli ST131 isolates than do the available comparable PCR assays, plus offer a novel sequence-based alternative to PCR. They may prove useful for molecular epidemiological studies, surveillance, and, potentially, clinical management.

## INTRODUCTION

Escherichia coli sequence type 131 (ST131), which arguably is the most epidemiologically successful multidrug-resistant E. coli lineage ever identified, emerged dramatically beginning in approximately 2000 to become a pandemic pathogen (1–5). ST131 now accounts for the greatest share of multidrug-resistant human extraintestinal E. coli infections worldwide (6–12).

**FIG 1:**
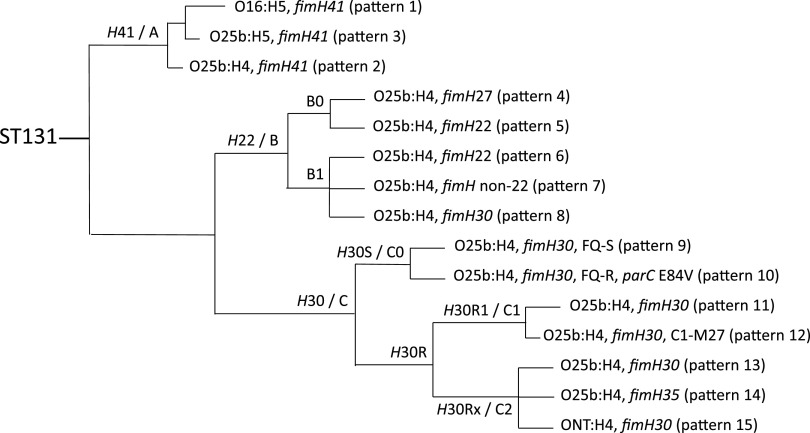
Schematic of the Escherichia coli ST131 phylogeny. Branch lengths are for illustrative purposes only and are not proportional. Clades with undefined branching order are shown as polytomies. Main branches are labeled selectively as to sublcone or clade (using alternative taxonomies); terminal branches are labeled selectively as to O:H serotype, *fimH* allele, fluoroquinolone (FQ) phenotype (FQ-S [susceptible], FQ-R [resistant]), *parC* genotype, and PCR pattern no.

ST131 exhibits a complex clonal structure, as elucidated by phylogenetic analyses based on whole genome sequencing (WGS) data (2, 3, 5, 13) (Fig. 1). The most extensively expanded ST131 component is a recently evolved sub-lineage that has been designated variously as *H*30R—reflecting its association with both fluoroquinolone resistance and the type-1 fimbrial adhesin allele *fimH30* (3)—and clade C, according to an alternate, trait-agnostic taxonomy (2, 5). Strains from *H*30R (clade C) typically exhibit the O25b somatic antigen and the H4 flagellar antigen.

*H*30R in turn has important subcomponents, including subclones *H*30R1 (clade C1) and *H*30Rx (clade C2) (2, 3, 5, 13) (Fig. 1). Although most *H*30R1 strains are cephalosporin-susceptible, some – especially within an emerging subclade designated M1-27, which was identified first in Asia and is expanding now in Europe and North America – carry *bla*_CTX-M-27_ or, less commonly, *bla*_CTX-M-14_ (14–16). By contrast, most *H*30Rx (clade C2) strains carry *bla*_CTX-M-15_, the distinctive characteristic that led to ST131's initial discovery (3, 17).

In addition to *H*30R, *H*30 also has a basal, fluoroquinolone-susceptible *H*30S component (clade C0), which may be an evolutionary precursor to the pandemic *H*30R subclone (2, 3, 5, 13) (Fig. 1). The evolutionary steps and fitness factors that led from this epidemiologically unsuccessful ancestor to the world-changing *H*30R lineage remain incompletely understood, despite considerable study (2, 10).

Apart from the dominant *H*30 clade, other ST131 subcomponents also have distinctive resistance phenotypes, epidemiological associations, *fimH* alleles, O types, and *fliC* (flagellar H antigen) alleles (Fig. 1). For example, the ancestral *H*41 subclone (clade A) is associated with trimethoprim-sulfamethoxazole resistance, younger patients, *fimH41*, and the O16 and H5 antigens (18). Likewise, the phylogenetically intermediate *H22* subclone (clade B), which typically is less extensively antimicrobial-resistant than the other ST131 subclones, is associated with food animals, including poultry and swine, where it can acquire antimicrobial resistance that subsequently transmits to humans (19). It is associated with *fimH22* and, like the *H*30 subclone, the O25b and H4 antigens (20, 21). Like other ST131 subsets, the *H*22 subclone has a complex substructure, definitions of which are evolving.

ST131's clear clinical and epidemiological importance, and its various distinctive sublineages, has motivated the development of PCR-based assays for use in screening and characterizing clinical, commensal, and environmental E. coli isolates (18, 21–26). These assays have become increasingly sophisticated and discriminating over time, in response to emerging understandings of the ST131 substructure (23). Ultimately, WGS-based phylogenetic analysis and accessory trait identification provides the most granular and robust characterization of E. coli isolates (2, 10, 27–29). However, given the as-yet limited widespread availability and affordability of WGS technologies for general epidemiological and clinical use, we sought here to devise an enhanced multiplex PCR-based assay for the detection and molecular characterization of important ST131 sublineages, accessible to any laboratory equipped for conventional endpoint PCR. Additionally, for those researchers with access to WGS, we also developed a WGS-based method that aimed to replicate *in silico* the multiplex PCR-based assay.

## RESULTS

### Specificity for ST131.

In the J. Johnson Laboratory the PCR assay correctly identified as non-ST131 all 146 negative control isolates, i.e., for them it did not yield any of the three possible ST131-specific amplicons: *mdh36*, *gyrB47*, or *trpA72*. Thus, for ST131 *per se* the assay's estimated specificity was 100% (95% CI, 97.5–100%).

### Subclade detection within ST131.

When the new assay was applied to the Price collection in the J. Johnson Laboratory, in comparison with the published WGS phylogeny (Supplemental material 1) it classified correctly by subclone all 105 ST131 strains (100% accuracy; 95% CI, 96.6% – 100%). Similarly, when it was applied to the Gordon collection in the D. Gordon laboratory, in comparison with an unpublished WGS phylogeny (Supplemental material 2) it classified correctly by subclone all 73 ST131 strains (100% accuracy; 95% CI, 95.1% – 100%).

In the J. Johnson Laboratory, the assay yielded 100% concordance with concurrently performed single or dual-target PCR assays for detecting the *fimH30* allele, the *H*30Rx clade, and the O25b and O16 *rfb* variants. It also yielded 100% accuracy (95% CI, 96.6–100%) for detecting the *parC* E84V mutation, as identified by WGS (with or without supplemental Sanger sequencing), among 5 ST131 isolates with and 46 without this *parC* allele.

### Assay stability.

In the J. Johnson Laboratory, after optimization the assay's performance was consistent across three different thermal cyclers: Eppendorf Mastercycler x50, Bio-Rad MyCycler, and Bio-Rad T1000. Notably, however, with the Bio-Rad T1000 a 1°C higher annealing temperature was required with all pools to avoid nonspecific amplification.

### Diversity of PCR profiles.

**FIG 2:**
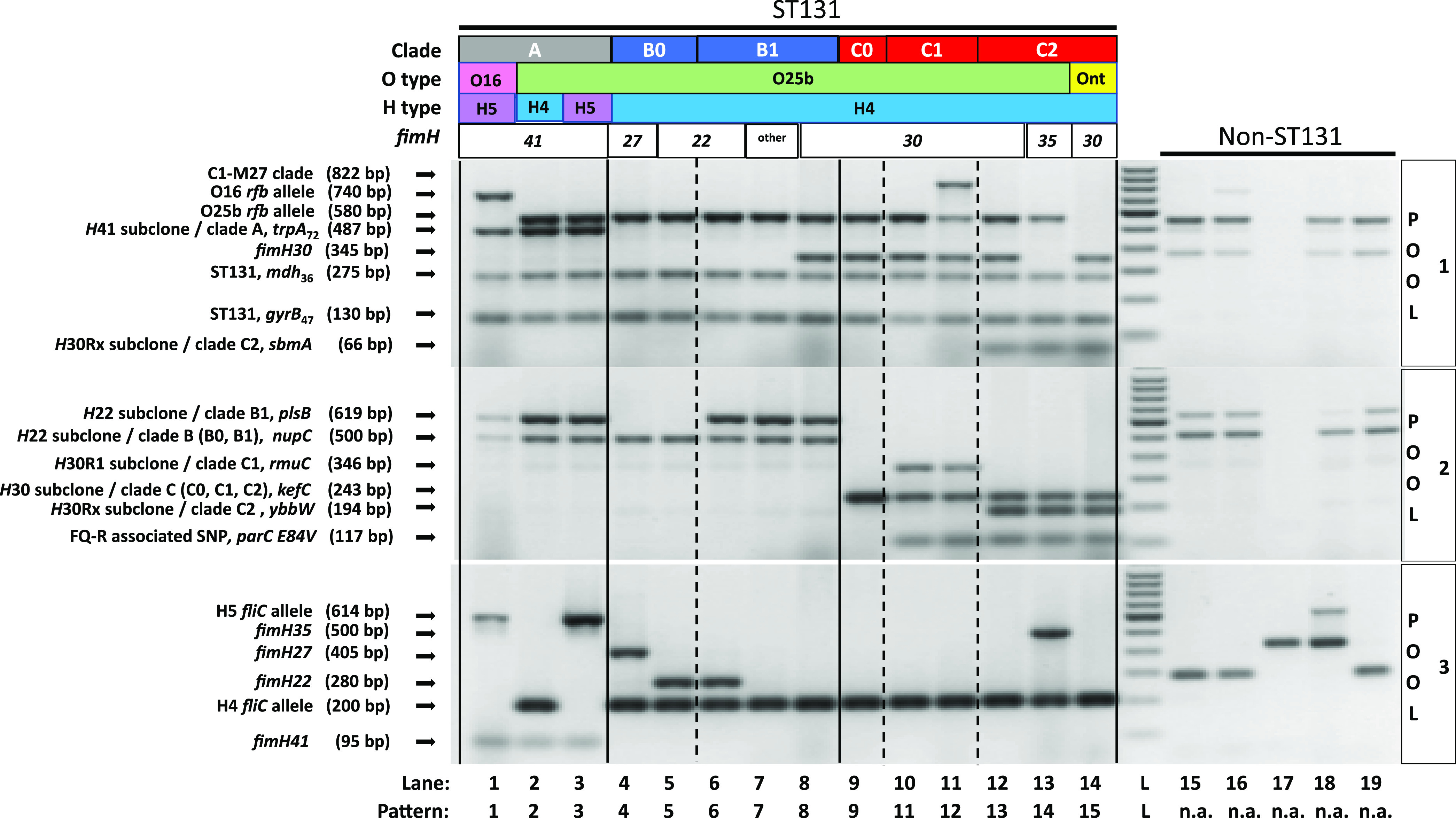
PCR profiles for primer pools 1–3 with representative ST131 and non-ST131 Escherichia coli isolates. Primer pools are listed to right of gel images (pool 1, top; pool 2, middle; pool 3, bottom). Lanes 1–14, representative ST131 isolates for 14 of the 15 PCR patterns observed with the new assay, as listed below image. (The missing pattern was observed only in the D. Gordon laboratory; the representative isolates was unavailable in the J. Johnson laboratory for inclusion in this image.) Lane L, 100 bp ladder. Lanes 15–19, diverse non-ST131 E. coli isolates from STs (lane no.) 10 (15), 73 (16), 95 (17), 144 (18), and 69 (19). Horizontal text boxes above gels list, from top to bottom, ST131 status; ST131 clade/subclade; O type; H type; and *fimH* allele. Labels to left of gels identify each band and its size in base pairs (bp). Phylogenetic subsets are labeled using alternate taxonomies.

Within the combined Price and Gordon collections (*n* = 179 isolates), the new assay identified 8 unique PCR profiles with just primer pool 1, whereas the addition of primer pools 2 and 3, and the 9-isolate Miscellaneous collection, increased this number to 15 (Table 1, Fig. 2). The 15 unique PCR profiles differed widely in frequency by collection (Table 2). The 8 most frequent profiles were encountered in both the Price and Gordon collections. Presence in only one—or neither—of these collections was limited to the 7 profiles with only one to three representatives each. These profiles included *H*30Rx:O-nontypeable (isolate JJ2449: Miscellaneous collection only), B0:*fimH22* (isolate JJ1897: Price collection only), B1:*fimH22*-negative (isolates JJ2016, ZH71, and JMI025: Price collection only); *H*35Rx/C2 (isolates JJ2643 and U004: Price collection only); A:O25b:H4 (isolates M685551, M652483 and BS488: Gordon collection only), A:O25b:H5 (isolate M670745: Gordon collection only), and C0:*parC* E84V (isolate BS448: Gordon collection only).

**TABLE 1:** Distinguishing features of 15 unique PCR patterns observed among the present Escherichia coli study isolates with the novel 3-tube multiplex ST131 PCR assay^*a*^

			ST131, subclones, clades										*fimH*					*rfb*		*fliC*		FQR
Pattern no.	ST131	Subclone/clade	*gyrB47*	*mdh36*	*trpA72*	*nupC* SNP	*plsB* SNP	*kefC* SNP	*rmuC* SNP	*ybmW* SNP	*sbmA* SNP	Prophage	*fimH22*	*fimH27*	*fimH30*	*fimH35*	*fimH41*	O16	O25b	H4	H5	*parC* E84V
1	+	*H*41/A	+	+	+	na^*b*^	na^*b*^	−	−	−	−	−	−	−	−	−	+	+	−	−	+	−
2	+	*H*41/A	+	+	+	na^*b*^	na^*b*^	−	−	−	−	−	−	−	−	−	+	−	+	+	−	−
3	+	*H*41/A	+	+	+	na^*b*^	na^*b*^	−	−	−	−	−	−	−	−	−	+	−	+	−	+	−
4	+	*H*22/B0	+	+	−	−	+	−	−	−	−	−	−	+	−	−	−	−	+	+	−	−
5	+	*H*22/B0	+	+	−	−	+	−	−	−	−	−	+	−	−	−	−	−	+	+	−	−
6	+	*H*22/B1	+	+	−	+	+	−	−	−	−	−	+	−	−	−	−	−	+	+	−	−
7	+	*H*22/B1^*c*^	+	+	−	+	+	−	−	−	−	−	−	−	−	−	−	−	+	+	−	−
8	+	*H*22/B1	+	+	−	+	+	−	−	−	−	−	−	−	+	−	−	−	+	+	−	−
9	+	*H*30S/C0	+	+	−	−	−	−	+	−	−	−	−	−	+	−	−	−	+	+	−	−
10	+	*H*30S/C0^*d*^	+	+	−	−	−		+	−	−	−	−	−	+	−	−	−	+	+	−	+
11	+	*H*30R1/C1, non-M27	+	+	−	−	−	+	+	−	−	−	−	−	+	−	−	−	+	+	−	+
12	+	*H*30R1/C1-M27	+	+	−	−	−	+	+	−	−	+	−	−	+	−	−	−	+	+	−	+
13	+	*H*30Rx/C2	+	+	−	−	−	−	+	+	+	−	−	−	+	−	−	−	+	+	−	+
14	+	*H*30Rx/C2^*e*^	+	+	−	−	−	−	+	+	+	−	−	−	−	+	−	−	+	+	−	+
15	+	*H*30Rx/C2^*f*^	+	+	−	−	−	−	+	+	+	−	−	−	+	−	−	−	+	+	−	+
n.a.^*b*^	−	non-ST131	+	−	n.a.^*b*^	n.a.^*b*^	n.a.^*b*^	n.a.^*b*^	n.a.^*b*^	n.a.^*b*^	n.a.^*b*^	n.a.^*b*^	n.a.^*b*^	n.a.^*b*^	n.a.^*b*^	n.a.^*b*^	n.a.^*b*^	n.a.^*b*^	n.a.^*b*^	n.a.^*b*^	n.a.^*b*^	n.a.^*b*^
n.a.^*b*^	−	non-ST131	−	+	n.a.^*b*^	n.a.^*b*^	n.a.^*b*^	n.a.^*b*^	n.a.^*b*^	n.a.^*b*^	n.a.^*b*^	n.a.^*b*^	n.a.^*b*^	n.a.^*b*^	n.a.^*b*^	n.a.^*b*^	n.a.^*b*^	n.a.^*b*^	n.a.^*b*^	n.a.^*b*^	n.a.^*b*^	n.a.^*b*^
n.a.^*b*^	−	non-ST131	−	−	n.a.^*b*^	n.a.^*b*^	n.a.^*b*^	n.a.^*b*^	n.a.^*b*^	n.a.^*b*^	n.a.^*b*^	n.a.^*b*^	n.a.^*b*^	n.a.^*b*^	n.a.^*b*^	n.a.^*b*^	n.a.^*b*^	n.a.^*b*^	n.a.^*b*^	n.a.^*b*^	n.a.^*b*^	n.a.^*b*^

a FQR, fluoroquinolone-resistant; SNP, single-nucleotide polymorphism; + and − signs, presence or absence of trait.

b n.a., not applicable: isolates testing PCR positive for *trpA72* (Pool 1) are assigned to clade O16/A and therefore results of PCR Pool 2 should not be considered as this pool is only designed to characterize the non-clade A types (clades B and C). (Although not applicable, clade A isolates do typically amplify the two clade B associated PCR amplicons in pool 2).

c PCR Pattern 7 is representative of a *fimH* allele type (*fimH94*) not detected by primers in this PCR assay.

d PCR Profile 10 retains the historical consensus subclade label “*H*30S/CO” as determined by *kefC* clade C PCR positive results and *rmuC* (C1) and *ybbW* (C2) negative PCR result. The *parC* E84V positive PCR and corresponding fluoroquinolone-resistant phenotype differentiate this profile type from Profile 9, representing the consensus *H*30S/C0 representative being *parC* E84V negative with a fluoroquinolone-susceptible phenotype.

e *fimH*35 variant of H30Rx/C2 subclade.

f O-type negative (as determined by classic serotype methods at the E. coli Reference Center (The Pennsylvania State University), and also reflected within assay, PCR negative for bothO25b and O16 PCR negative variant of *H*30Rx/C2 subclade).

**TABLE 2:** Multiplex PCR profile types, associated traits, representative strains, and number per study collection

					Collection		
PCR type	Clade	O:H type	*fimH* allele	Representative strain	Price (*n* = 105)	Gordon (*n* = 73)	MVAST (*n* = 20/90)^*a*^
1	A	O16:H5	41	MVAST020	7	11	5
2	A	O25b:H4	41	BS488	0	3	0
3	A	O25b:H5	41	M670745	0	1	0
4	B0	O25b:H4	27	H17	1	1	0
5	B0	O25b:H4	22	JJ1897	2	0	0
6	B1	O25b:H4	22	JJ1969	28	21	1
7^*b*^	B1	O25b:H4	94^*b*^	ZH071^*b*^	3	0	0
8	B1	O25b:H4	30	G199	1	3	0
9	C0	O25b:H4	30	CD306	4	2	0
10^*c*^	C0^*c*^	O25b:H4	30	BS448^*c*^	0	1	0
11^*d*^	C1	O25b:H4	30	JJ2193	32	11^*d*^	10
12	C1-M27	O25b:H4	30	U024	1	7	1
13	C2	O25b:H4	30	JJ1886	24	12	3
14	C2	O25b:H4	35	U004	2	0	0
15^*e*^	C2	ONT^*e*^:H4	30	JJ2449^*e*^	0^*e*^	0^*e*^	0^*e*^

a From the (*n* = 90) all-comers MVAST collection (Table Y) only the 20 ST131 isolates are shown here.

b The *fimH94* allele is not part of the *fimH22* allele complex and is not detected by this multiplex PCR assay. Thus, PCR profile type #7 lacks any *fimH* allele band.

c Isolate BS448, although from clade C0, has the typical *gyrA*/*parC* haplotype of fluoroquinolone-resistant *H*30R (C1, C2) isolates, and with primer pool 1 produces the expected *parC* amplicon.

d Two of the 11 isolates that represent PCR profile type #11 have *fimH99* (which exhibits 1 single-nucleotide polymorphism difference versus the *fimH30* allele, not in the primer region).

e ONT, O-nontypeable. This profile uniquely lacks both the O25b and O16 *rfb* amplicons. The only known representative isolate, JJ2449, is from the “Misc.” collection (Table 4), not one of the collections listed here.

### Comparison with established PCR assays among fresh clinical E. coli isolates.

To simulate a clinical diagnostics application, 90 consecutive fresh clinical isolates from the MVAMC microbiology laboratory were tested in parallel using (i) the novel assay and (ii) established single or dual-target PCR assays. The assays were compared for their results regarding ST131 status, *fimH30*, *H*30Rx, and the O25b and O16 *rfb* variants. The compared assays yielded 100% concordant results (95% CI, 96.4% - 100%), jointly identifying as ST131 the same 20 isolates, of which 5 were O16/A (all non-*H*30) and 15 were O25b (1 non-*H*30/B and 14 *H*30/C, including 3 *H*30Rx/C1). This collection's 20 ST131 isolates exhibited the 5 most common ST131-associated PCR patterns encountered in the Price and Gordon collections (Table 2).

Additionally, in this testing of fresh clinical isolates the new multiplex assay augmented the conventional assays in several ways. Specifically, it confirmed the 5 O16/A ST131 isolates by both traditional *rfb* O16 and the *trpA* clade A-specific amplicon (negative in all O25 ST131 isolates) and confirmed the 3 *H*30Rx/C2 isolates based on clade-specific SNPs in both *sbmA* and *ybbW*. It also uniquely assigned one of the 14 *H*30/C isolates to the (recently described) C1-M27 clade, assigned the single non-*H*30 O25b isolate to the B1 subclade, showed that this isolate had the *fimH22* allele, identified all O16 isolates as having *fliC* H5 and *fimH41*, and identified all O25 isolates as having *fliC* H4.

### Portability assessment.

For a formal assessment of the new assay's portability across laboratories it underwent independent, blinded testing in the T. Johnson Laboratory, which is geographically removed from the originating (J. Johnson) laboratory. After an initial optimization phase involving known positive and negative controls, plus open between-laboratory communication regarding technical issues, blinded validation testing was done using a panel of 18 coded test isolates (12 ST131, 6 non-ST131) (Supplemental material 3). The blinded testing correctly identified the isolates' ST131 status, but initially yielded discrepancies regarding their extended PCR profiles. Assay modifications were implemented to address these discrepancies, including (i) length adjustments in one *fimH22* primer and one *ybbW* primer and (ii) more explicit instructions for gel interpretation. These modifications yielded 100% accurate assay results, without a need to adjust annealing temperatures.

### ST131Typer performance assessment.

The overall accuracy of the (WGS-based) ST131Typer as a surrogate for the PCR assay, based on the 255 isolates tested, was 87.8% (95% CI, 83.2–91.6%). However, performance varied greatly by endpoint. Specifically, ST131Typer correctly identified all 78 negative control isolates as non-ST131 (100% specificity; 95% CI, 95.4–100%) and correctly classified 171 of the 173 ST131 strains by subclade (97.7% accuracy; 95% CI, 94.3–99.4%). By contrast, ST131Typer yielded the same PCR profile as the *in vitro* extended multiplex PCR assay for only 142 (82.5%; 95% CI, 76.1–87.8%) of the 173 ST131 strains.

Analysis of the 31 total instances where ST131Typer results did not match the PCR profile showed that 19% (6/31) were due to ST131Typer profile misclassifications and 81% (25/31) to unknown profile classifications. Supplemental material 5 lists the strains with a discrepancy, their placement in the phylogeny, their associated characteristics, and the specific discrepancies. (For the remaining 142 strains, ST131Typer and PCR agreed for all markers and classifications.) All 31 ST131Typer errors (one per strain, for 31 strains) were caused by inability to identify a single required target sequence, specifically the *rfb* O25b variant (16 strains), *fliC* H4 (7 strains), *kefC* (4 strains), and *fimH22*, *fimH30*, *fimH40*, and prophage marker (1 strain each).

Potentially explaining nearly all missing *rfb* O25b targets (15/16), and all four missing *kefC* targets, were highly fragmented and incomplete genome assemblies, with the forward and reverse primer sequences on different contigs, or one or both not detected. Indeed, N50 values (a common metric for genome assemblies; smaller values indicate greater fragmentation) were considerably lower for assemblies with incorrect, versus correct, PCR profile classifications (Mean ± SD: 58,963 ± 57,937 versus 224,646 ± 564,985). When use as a supplemental typing method CGE’s SerotypeFinder identified the missing *rfb* O25b variant in 15 of the 16 strains where ST131Typer missed it. However, match identities were <100%, and high-scoring BLAST alignments typically shorter than the reference *rfb* O25b sequence.

Additional instances of mismatch between ST131Typer and the PCR assay were potentially from sequence variation in the forward or reverse primer regions. This was particularly evident with the missing *fliC* H4 and *fimH* target sequences, where the primer regions frequently included one or more SNPs. As for the *rfb* O25b target, SerotypeFinder identified the *fliC* H4 variant in all these samples, but with match identities <100% and high-scoring BLAST alignments shorter than the reference *fliC* H4 sequence. Likewise, for the three samples with a missing *fimH* allele per ST131Typer, CGE’s FimTyper identified *fimH* alleles that represented minor (1–2 nucleotide) variants of the alleles detected by PCR.

## DISCUSSION

In this study, we developed and extensively validated a novel 3-pool, multiplex PCR assay—and a WGS-based, *in silico* analog of it—for identifying and characterizing E. coli ST131 isolates. The PCR assay demonstrated an excellent ability to distinguish E. coli ST131 from other E. coli, to classify ST131 isolates by subclonal type (in comparison with phylogenomic analyses and established PCR-based assays), and to characterize the isolates for key accessory traits, including relevant *fimH* and *fliC* alleles, the O25b versus O16 *rfb* variants, and a fluoroquinolone resistance-associated *parC* SNP. The assay also was portable across laboratories and performed well with fresh clinical isolates. Thus, this novel PCR assay should prove useful for molecular epidemiological studies and, potentially, clinical diagnostics. The *in silico* version of the assay, ST131Typer, accurately distinguished E. coli ST131 from other E. coli, and classified ST131 isolates accurately into subclades. However, its ability to replicate the PCR assay's full profile patterns was impeded by incomplete genome assemblies and minor sequence variation.

Several PCR-based assays have been described previously—including by some of the present authors—for detecting ST131 *per se*, key ST131 subsets, and various relevant accessory traits (18, 21–26). The most extensive of these assays differentiates ST131 from non-ST131 isolates; resolves ST131 clades A, B, and C; and, within clade C, resolves subclades C1-non-M27, C1-M27, C2, and “C, not C1 or C2” (23). Additionally, in that assay the presence of an ST131 amplicon but absence of all clade/subclade amplicons identifies an intermediate branch between clades B and C. The present novel assay supersedes that and other previous assays by detecting multiple subsets and identifying relevant accessory traits within all clades and subclades.

The present multiplex assay, with its extensive range of targets, yielded several novel findings. Specifically, within clade A it identified uncharacteristic O25b-associated variants, in addition to the expected O16-associated variants, and separated these according to *fliC* allele. Comparison with a SNP-based phylogeny (Supplemental material 2) showed that this variation in accessory traits within clade A was phylogenetically distributed, with the “atypical” variants occupying a basal position. This suggested that the O25b *rfb* allele and *fliC* H4 were possibly ancestral within clade A and, hence, perhaps within ST131 as a whole.

Additionally, within clade C2 the new assay differentiated isolates with (uncharacteristic) *fimH35* from those with (characteristic) *fimH30*. Likewise, within subclades B0 and B1 it differentiated isolates with (uncharacteristic) *fimH27* from those with (characteristic) *fimH22*, or neither of these. It also identified within clade C0 an isolate (BS448) that uniquely exhibited the distinctive fluoroquinolone resistance-associated *parC* SNP (a251t = E84V) that typifies clades C1 and C2, that is, *H*30R (3, 30). Notably, according to WGS analysis (not shown) that isolate also had the three other *H*30R-associated fluoroquinolone resistance-conferring replacement mutations in *gyrA* (c248t = S83L; g259Aa = D87N) and *parC* (g239t = S80I) (30), suggesting that it may be an immediate ancestor to the *H*30R pandemic lineage. Finally, absence of the O25b and O16 *rfb* variants in one *H*30Rx/C2 isolate defined the isolate as O-nontypeable by the present assay; its O-nontypability was confirmed at a reference laboratory (E. coli Reference Center, The Pennsylvania State University) by conventional broad-range O serotyping. Whether this isolate's distinctive PCR profile represents a new O-negative *H*30Rx subtype or is unique to this particular isolate remains to be determined.

Although the novel assay was designed around three primer pools (i.e., three PCRs), the individual pools can be used separately, including in a hierarchical manner. For example, generic E. coli isolates can be screened initially using pool 1, which in addition to detecting ST131 *per se* can resolve 8 different subclonal profiles. Isolates so identified as ST131 can then be further characterized using pools 2 and 3, which offer refined subclone/confirmatory discrimination and can identify additional accessory traits, including *H*30R's hallmark fluoroquinolone resistance-associated *parC* SNP (3, 30).

When validated using an extensive panel of positive and negative control strains, the assay yielded 100% accuracy in comparison with external reference standards, including WGS analyses or individual PCR results. Additionally, it was portable across laboratories. Notably, however, in each new laboratory some optimization was needed. Thus, when the assay is established in additional laboratories further optimization may be needed to ensure the expected performance with relevant control strains.

The assay has notable limitations. First, other than the hallmark fluoroquinolone resistance-associated mutation in *parC*, it lacks resistance gene targets, including the various ST131-associated *bla*_CTX-M_ variants and carbapenemase-encoding genes, for which supplemental assays would be needed. Second, its amplicon profiles are complex, potentially posing interpretive challenges. Adherence to detailed instructions and use of the provided interactive data entry spreadsheet can help. Third, the particular sublineages and associated traits included in the assay represent only a fraction of the ever-expanding number of possible targets and were selected somewhat arbitrarily. For example, the assay addresses minimally the recently appreciated clonal diversity within the *H*22/clade B lineage (19, 20). The present version of the assay represents a compromise between comprehensiveness and practicality; future iterations could address emerging markers of interest. Fourth, assay optimization will be needed in new user laboratories, possibly obliging communication with the developers.

Due to the increasing demand for sequence-based analytical tools, we also developed ST131Typer as a WGS-based alternative to the *in vitro* PCR assay. ST131Typer was highly accurate in distinguishing E. coli ST131 isolates from other E. coli and in classifying them into ST131 subclades but was only moderately accurate in replicating the PCR assay's profiles. The main issue appeared to be fragmented or incomplete input assemblies, which, in turn, were likely caused by poor quality reads and/or insufficient genome sequence coverage. Thus, the accuracy of ST131Typer's results depends on sequence and assembly quality. Currently, sufficient next-generation sequencing coverage and high-quality genome assembly are still major challenges in some research and clinical settings. Analysis of WGS data often requires considerable computational resources and a bioinformatics skill set, and associated costs can be prohibitive if large numbers of samples are processed. As such, the novel *in vitro* PCR assay presented here may be a useful, even preferable, ST131 characterization tool for general epidemiological and clinical use, especially in resource-limited settings.

In conclusion, this novel PCR assay, consisting of 3 multiplex primer pools, can rapidly and specifically distinguish ST131 from other E. coli and characterize ST131 isolates according to multiple phylogenomic subclones and accessory trait, yielding 15 distinct trait profiles with the present reference strains. ST131Typer, a novel web-accessible, WGS-based tool, provided a reasonably accurate *in silico* surrogate for the PCR assay, especially when supplemented with CGE-supported subroutines. Pending the wider availability, affordability, and user-friendliness of WGS and the associated bioinformatics approaches, this PCR assay should prove useful for epidemiological studies and, potentially, clinical diagnostics.

## MATERIALS AND METHODS

### Assay design and primers.

The assay was designed to identify two different types of entities: phylogenetic subsets (seven total) and accessory traits (four distinct traits; 10 total variants thereof). The seven phylogenetic subsets of interest (alternate taxonomy labels in parentheses) included the three major ST131 subdivisions, i.e., subclones *H*41 (clade A), *H*22 (clade B), and *H*30 (clade C), and components thereof. These components were, within the *H*22 subclone, subclade B0 (versus the rest of subclade B, hereafter referred to as B1); within the *H*30 subclone, subclones *H*30S (C0), *H*30R1 (C1), and *H*30Rx (C2); and within subclone *H*30R1, the (*bla*_CTX-M-27_-associated) C1-M27 subclone. The accessory traits of interest included diverse ST131-associated variants of four key genes: *fimH* (type-1 fimbriae adhesin) (31), *fliC* (flagellar H antigen), *rfb* (O antigen), and *parC* (topoisomerase), specifically the (*H*30R1 and *H*30Rx)-associated E84V *parC* variant that is associated with higher fluoroquinolone MICs than those of other fluoroquinolone-resistant strains (30, 32).

Relevant published primers were used as-is or were modified as needed for optimized performance and compatibility with other primers. Novel primers were designed based on published target sequences and were optimized – for sensitivity, specificity, amplicon size, and compatibility with other primers – based on *in vitro* performance against relevant positive and negative control strains, both as individual primer pairs and in multiplex pools. Amplicon specificity was assessed by comparing the observed product size with the expected product size for the particular target, not by sequencing. Primer sensitivity and specificity were assessed based on ability to amplify a product of the expected size with positive control strains but not negative control strains.

The final assay comprised 36 total primers: 22 were novel and 14 were previously published (Table 3). These primers target allele-specific single-nucleotide polymorphisms (SNPs) or nucleotide sequences in *mdh36* and *gyrB47* (ST131-specific), the O16 and O25b *rfb* variants, 5 *fimH* alleles, 2 *fliC* alleles, the fluoroquinolone resistance (FQR)-determining region of *parC* and selected other clade-specific genes or alleles thereof. The subclade markers (associated target clade/subclade) include *trpA72* (clade A), *plsB* (clade B1), *nupC (*clade B, including subclades B1 and B0), *kefC* (clade C, including subclades C0, C1, and C2), *rmuC* (subclade C1), *ybbW* and *sbmA* (subclade C2), and a prophage marker (C1-M27 subclade).

**TABLE 3:** Primers targets and primer sequences for the three multiplex pools^*a*^

Pool	Target	Target significance^*b*^	Product size (bp)	Forward (for) and reverse (rev) primer sequence (5′–3′)	Conc. (*u*M)	Reference
1	Prophage	C1-M27 clade	822	(For) TTACTCCGACTATGCGTTCAC	0.25	(16)
				(Rev) CTTCTTCGCTGGCAACTTCT	0.25	This study
	*rfb* *O16* allele	O16 antigen	732	(For) GAAGGTGTTACCACCATCAATG	0.25	This study
				(Rev) CAGGATCATTTATGCTGGTACG	0.25	(44)
	*rfb* *O25b* allele	O25b antigen	557	Same as O16 forward primer	n.a.^*c*^	This study
				(Rev) ATGCTATTCATTATGCGCAGC	0.25	(44)
	*trpA72*	O16 subclone/clade A	487	(For) CTTTTGCGGCGGGAGTA	0.25	This study
				(Rev) GAAATCGCGCCCGCAGT	0.25	(18), this study
	*fimH30* allele	*fimH30* allele complex	350	(For) GCCAATGGTACCGCTATT	0.25	(30)
				(Rev) GCTTTAATCGCCACCCCA	0.25	(30)
	*mdh36*	ST131-specific SNP	273	(For) TTAACGTTAACGCCGGT	0.4	(22)
				(Rev) TGGTAACACCAGAGTGACCA	0.4	(22)
	*gyrB47*	ST131-specific SNP	138	(For) CTGCGCGATAAGCGCGAC	0.15	(22)
				(Rev) AATACCGTCTTTTTCGGTGGAA	0.15	(22)
	*sbmA*	*H*30Rx subclone/clade C2	64	(For) GCTGCCTACCGTAAAGAA	0.25	This study
				(Rev) CCGTAGGCGGCGTCA	0.25	This study
2	*plsB*	*H*22 subclone/clade B1	628	(For) AGAATTCCGGCGCGTTG	0.4	This study
				(Rev) CGCGAGCAGTTAACCG	0.4	This study
	*nupC*	*H*22 subclone/clades B0, B1	498	(For) TCCCGCAATCGTATGTACACC	0.4	This study
				(Rev) ATCCATCATCGCAACGAAC	0.4	This study
	*rmuC*	*H*30R1 subclone/clade C1	353	(For) TATCGCGTCGGTGCGTAAC	0.25	This study
				(Rev) CCTGCGCTTTGTCGAGAT	0.25	This study
	*kefC*	*H*30 subclone/clade C (C0,C1,C2)	238	(For) CGCAAATGGGCCGCAGA	0.25	This study
				(Rev) GCACTAAACACTTCCCGCA	0.25	This study
	*ybbW*	*H*30Rx subclone/clade C2	188	(For) GTTGCGGTCTGGGCA	0.1	This study
				(For) TCCAGCACGTTCCAGGTG	0.1	This study
	*parC*	E84V amino acid substitution	107	(For) GGCGATATTGCCTGTTATGT	0.25	This study
				(Rev) TTCGGATCGTCCGGCGCC	0.25	This study
3	*fliC* H5 allele	H5 flagellar antigen	614	(For) CGTTCAAGCGACGACCGGG	0.4	This study
				(Rev) ATGTTACATATCCCGCAGCCGA	0.4	This study
	*fimH35* allele	*fimH35* allele complex	500	(For) CTGTAAAACCGCCAATGGTACA	0.4	This study
				(Rev) TGACATCACGAGCAGAAACATCG	0.4	This study
	*fimH27* allele	*fimH27* allele complex	417	(Same as *fimH22* forward)	n.a.^*c*^	This study
				(Same as *fimH35* rev)	n.a.^*c*^	This study
	*fimH22* allele	*fimH22* allele complex	279	(For) TGGGGCAAAACCTGGTCGTG	0.2	This study
				(Rev) CAGCTTTAATCGCCACTCCC	0.2	This study
	*fliC* H4 allele	H4 flagellar antigen	199	(For) GGCGAAACTGACGGCTGCTG	0.08	(45)
				(Rev) ACCAACAGTTACCGCCGC	0.08	(45), this study
	*fimH41* allele	*fimH41* complex	92	Same as *fimH35* forward	n.a.^*c*^	This study
				(Rev) TTTGCCCCACATTCACGGCG	0.2	This study

a For, forward; Rev, reverse; Conc., concentration.

b Phylogenetic subsets are labeled according to both the Price et al. taxonomy (which uses the term “subclone”) ref and the Petty et al. taxonomy (which uses the term “clade”) ref. In *fimH*-based subclone designations the H is italicized, to imply “*fimH*,” and avoid confusion with flagellar H antigens.

c n.a., not applicable: shared primers are used in combination with unique primers to amplify different PCR targets. The shared primer should be added only once, in the concentration first specified.

The primers were split into three multiplex reaction pools based on band size and annealing temperature (Table 3). Pool 1 included primers specific for ST131 *per se*; its subclades *H*41-O16 (clade A), *H*30Rx (clade C2), and C1-M27; the *fimH*30 allele; and the O16 and O25b *rfb* variants. Pool 2 included primers specific for subclades *H*22 (clade B), B1, *H*30 (clade C), *H*30R1 (clade C1), and *H*30Rx (clade C2: using a different target than pool 1), plus the E84V *parC* mutation. Pool 3 included primers specific for the H4 and H5 *fliC* alleles and the *fimH22*, *fimH35*, and *fimH41* allele complexes, with allele complex defined here as the type allele plus minor variants thereof.

### PCR conditions.

The three primer pools were used in separate PCRs and were tested on different thermal cyclers. A hot-start *Taq* polymerase was used (GoTaq Hotstart, Promega) with the supplied buffer, adjusted to contain either 4 mM MgCl_2_ (pools 1 and 2) or 2 mM MgCl_2_ (pool 3.) The cycle routines were as follows: pool 1, 95°C 2 min; 30 cycles each (94°C 15 s, 65°C 1 min 15 s), 72°C 2 min; pool 2, 95°C 2 min; 34 cycles each (94°C 15 s, 67°C 45 s), 72°C 2 min; and pool 3, 95°C 2 min; 38 cycles each (94°C 15 s, 1 min 15 s 65°C), 72°C 2 min. Reactions were then held at 12°C. Amplicon bands were resolved by 2% agarose gel electrophoresis and ethidium bromide staining.

### Strain sets and phylogenetic analysis.

Seven strain sets (432 total isolates) were used for assay derivation, validation, and portability assessment (Table 4). The individual strain sets and how they were used here are described below.

**TABLE 4:** Strain sets used for assay derivation, validation, and portability assessment^*a*^

Name	No. isolates	Nature of collection	Available metadata	How used	Which lab(s)	Ref.
Price	105	Diverse ST131 isolates	WGS, phylogeny, accessory traits	Derivation, validation	JJ	(3)
Gordon	73	Diverse ST131 isolates	WGS, phylogeny, accessory traits	Derivation, validation	DG	n.a.
MVAST	90	Consecutive clinical isolates	Single-target PCR results	Validation for clinical use	JJ	n.a.
ECOR	72	Reference E. coli isolates	WGS, *fimH* allele, ST	Non-ST131 neg. controls	JJ	(33)
BUTI	66 (65^*b*^)	Historical urosepsis isolates	WGS, *fimH* allele, ST	Non-ST131 neg. controls	JJ	(34, 35)
Misc.	9^*c*^	Extra STs, PCR type, phylogroup	WGS and/or ST; O:H serotype	ST131/non-ST131 controls	JJ	n.a.
Test	19^*d*^	Representative type strains	Novel PCR assay results	Portability assessment	JJ, TJ	n.a.

a lab, laboratory; Ref., reference; WGS, whole genome sequencing; JJ, James Johnson; DG, David Gordon; ST, sequence type; TJ, Timothy Johnson; n.a., not applicable (i.e., unpublished or multiple sources); Misc., miscellaneous.

b This 66-isolate collection contains one ST131 isolate and 65 non-ST131 isolates.

c Includes 8 non-ST131 isolates, from phylogroups A (ST617), B2 (ST1193), D (ST38), E (ST11), F (ST354), and G (ST 117, ST174), and 1 ST131 isolate (clade C2; *fimH*30; non-O25b/non-O16).

d Selected deliberately as 12 type-representative ST131 isolates and 7 phylogenetically diverse non-ST131 isolates, from phylogroups A (ST10), B2 (ST12, ST73, ST95, ST144, ST1194), and D (ST69).

### Non-ST131 controls.

The negative controls were 146 phylogenetically diverse non-ST131 E. coli isolates, including 138 from two published collections with available WGS data and eight supplemental isolates (assigned to the Miscellaneous collection) representing additional STs not found in these two collections (Table 4). The two published collections included the E. coli Reference (ECOR) collection (*n* = 72) (33) and a set of bloodstream isolates from patients with bacteremic urinary tract infection (*n* = 66 isolates) (34, 35). The eight supplemental isolates were from diverse-source collections in the J. Johnson Laboratory. Collectively, the negative controls included all major E. coli phylogroups (A, B1, B2, C, D, E, F, and G) (36, 37), each represented by from 3 to 30 ST-*fimH* types (31) (Table 5), giving 97 total unique non-ST131 ST-*fimH* types (Table 5).

**TABLE 5:** Distribution of non-ST131 Escherichia isolates (*n* = 146) by phylogenetic group^*a*^, ST^*b*^, and ST-*fimH* allele combination^*c*^

Phylo-group^*a*^	Isolates (no.)	Unique STs^*b*^ (no.)	Unique ST^*b*^-*fimH* allele combinations^*c*^ (no.)	Specific STs included
A	29	16	24	10, 43, 44, 45, 46, 47, 48, 49, 50, 51, 52, 63, 65, 77, 87, 617
B1	16	13	13	53, 54, 55, 56, 58, 67, 75, 84, 85, 86, 88, 89, 101
B2	60	19	30	12, 14, 73, 74, 76, 78, 79, 80, 81, 82, 83, 95, 127, 144, 550, 625, 998, 1193, 4862
C	8	3	3	23, 88, 410
D	13	12	13	38, 66, 68, 69, 70, 71, 72, 106, 393, 405, 501, 598
E	9	4	5	11, 57, 61, 64
F	9	6	7	59, 60, 62, 354, 618, 648
G	2	2	2	117, 174
Any	146	75	97	All of above

a Phylogroup defined by Clermont Typer (*in silico*, using whole genome sequencing data) (46) or multiplex PCR (47).

b STs, sequence types, as defined using Achtman multilocus sequence typing (MLST) (https://enterobase.warwick.ac.uk/species/ecoli/allele_st_search).

c ST-*fimH* allele combinations provide sub-ST discrimination.

### ST131 controls.

The positive controls were 178 diverse genome-sequenced ST131 isolates (Table 4). These included 105 isolates from Price et al. (3) (hereafter, the Price collection), 73 isolates from the D. Gordon laboratory (37) (hereafter, the Gordon collection), and one isolate with a unique ONT:H4 serotype (assigned to the Miscellaneous collection). The Price and Gordon collections, as described in greater detail below, were selected deliberately from larger private collections of ST131 isolates to provide a broad range of phylogenetic subsets, sources, and accessory trait profiles.

The Price collection (*n* = 105) was used in the first WGS-based phylogenetic analysis of ST131 (3) and was the main component of subsequent studies that extended this analysis with additional strains and bioinformatics approaches (2, 10, 13). These analyses largely concurred regarding the sub-ST clonal structure of ST131, but used alternate taxonomies for the observed cladal subsets, one based on clade-associated accessory traits (i.e., *fimH* alleles, O types, and antimicrobial resistance), the other on a (trait-agnostic) alpha-numeric system. According to the latter taxonomy, the Price collection is distributed as follows (no. isolates per clade or subclade): 7 A, 3 B0, 32 B1, 4 C0, 32 C1, 1 C1-M27, and 26 C2 (Supplemental material 1).

The Gordon collection (*n* = 73), which represents diverse sources, was distributed as follows in an unpublished core genome, SNP-based phylogeny (no. isolates, clade or subclade [per alpha-numeric taxonomy]): 15 A, 1 B0, 24 B1, 3 C0, 11 C1-non-M27, 7 C1-M27, and 12 C2 (Supplemental material 2). For this phylogeny, the SNP data were derived using PARSNP (https://github.com/marbl/parsnp), with recombination detection/filtration, after which RAxML and PhyML trees were generated using TOPALi v.2 (http://www.topali.org/). Sequence-based results for this collection also were available for predicted O:H serotype, *fimH* allele, and fluoroquinolone resistance-associated *gyrA/parC* mutations. These isolates underwent PCR-based testing in the Gordon laboratory, using the present novel assay according to protocols provided by the (source) J. Johnson Laboratory.

For the Price and Gordon collections, subclone/clade assignments (which were used as the reference standard for assessing the multiplex PCR assay) were based primarily on isolates' position within the corresponding core genome SNP-based phylogeny, for which subclone/clade identity was apparent from (i) comparisons with published ST131 phylograms and (ii) the consensus characteristics of a given subclone or clade's constituent isolates. For the several strains that were placed ambiguously in the phylograms, i.e., at the junction of the *H22* and *H*30 subclones (clades B and C) or of the *H*30S and *H*30R subclones (C0 and C1/C2), subclone/clade assignment was resolved based on presence/absence of subclone/clade-specific genomic markers, as detected by WGS. Specifically, membership in the *H*30S subclone (clade C0) was defined by placement in the phylogeny within or immediately basal to the *H*30 subclone (clade C), but basal to the *H*30R1 and *H*30Rx subclones (clades C1 and C2), plus (according to WGS) containing the inclusive *H*30 subclone (clade C) marker *kefC* but lacking the markers for subclones *H*22 (clade B: *nupC*), *H*30R1 (C1: *rmuC*), and *H*30Rx (C2: *ybbW* and *sbmA*) (Table 3).

### Fresh clinical isolates.

Assay performance also was assessed in real time, using fresh clinical isolates. For this, 90 consecutive clinical E. coli isolates were retrieved over a 3-month span from the Minneapolis VA Medical Center clinical microbiology laboratory and tested in parallel using both the novel assay and established single-plex PCR assays for the detection of ST131, the O25b and O16 *rfb* variants, the *fimH30* allele, and the *H*30Rx/C2 subclone (38).

### Independent assay validation and blinded testing.

For a formal validation of the novel PCR assay in a laboratory other than that in which it was developed, an unmasked set of ST131 and non-ST131 E. coli isolates was provided to the T. Johnson Laboratory (University of Minnesota), along with the recommended assay protocol. In the T. Johnson Laboratory, the assay was done using a similar DNA extraction method (boiled lysates of overnight broth-grown bacteria) and similar reagents (including GoTaq Hotstart, Promega) as in the J. Johnson laboratory, but using a different type of thermal cycler (MyCycler, Bio-Rad, versus Mastercycler x50, Eppendorf). PCR products were separated in 2% agarose gels with ethidium bromide for visualization with UV light. Annealing temperatures and cycle numbers were adjusted as needed to achieve amplicon profiles for each pool similar to those shown in the provided protocol.

Once the assay was established in the T. Johnson Laboratory, a test set of 12 randomly assorted unique ST131 and 7 non-ST131 isolates (Supplemental material 3) was analyzed in a blinded manner. Results from the 3 multiplex pools were assessed for presence/absence of amplicons of the expected size for the particular pool. Data were entered into an interactive Excel file containing integrated interpretive algorithms (provided by the J. Johnson Laboratory: Supplemental material 4), which generated presumptive ST131 subclade assignments based on the binary amplicon data.

### ST131Typer description and performance assessment.

To create an *in silico* version of the extended multiplex PCR assay that utilizes WGS data, a command-line script was written to extract target sequences via the 20 primer pairs described in Table 3. Because the goal was to replicate *in silico* the *in vitro* multiplex PCR assay, and because the multiplex PCR assay infers amplicon specificity based strictly on amplicon size, the *in silico* version does so as well. To run an analysis, the user calls the bash script *ST131Typer.sh*, which accepts two mandatory arguments: a single FASTA/multi-FASTA assembly file or a directory of multiple FASTA assembly files, and the user-defined output directory. Amplicons are then identified in each input assembly using the SeqKit software’s *seqkit amplicon* function (39) and the provided tab-separated file of primer pair sequences. A valid amplicon is identified only if (i) 100% sequence identity is found between the primers and the input assembly sequence and (ii) both forward and reverse primer sequences are found on the same contig. No sequence analysis is done of the amplicon except for the primer-binding regions. Amplicon sequences greater than or less than 5% of the expected amplicon size are flagged in the final report with asterisks.

The ST131 PCR profile is then determined based on the same presence/absence patterns of subclone/clade-specific genomic markers as used in the *in vitro* extended multiplex PCR assay. If the target sequence for either *mdh36* or *gyrB47* is missing, the assembly is flagged as “non-ST131” and the remainder of the presence/absence amplicon pattern is ignored. If the *mdh36* and *gyrB47* targets are both present, but the resulting presence/absence amplicon pattern is not one of the PCR profiles described in Results, the assembly is characterized as unknown. If O-type, H-type, and/or *fim*H allele cannot be determined, the final report includes a warning message and recommends using additional typing tools such as the Center for Genomic Epidemiology’s (CGE's) SerotypeFinder (40) and FimTyper (41). The single output directory includes both a tab-separated final report summarizing (i) the results of all input assemblies and, for each assembly, (ii) the *seqkit amplicon* output, in BED format. The ST131Typer script, associated data files, and user instructions are freely available at the following GitHub repository: https://github.com/JohnsonSingerLab/ST131Typer.

To assess the accuracy of ST131Typer, all isolates used for the extended multiplex PCR assay derivation and validation, and for which WGS data were publicly available, were used (*n *=* *255). This included 78 non-ST131 E. coli isolates (primarily from the ECOR collection) and 177 phylogenetically diverse ST131 isolates (primarily from the Price and Gordon collections). Assemblies were available in EnteroBase (42) for most isolates. For the remaining isolates, Illumina paired-end reads were downloaded from NCBI’s Short Read Archive database and processed using the Shovill pipeline with the SPAdes assembler, adaptor trimming enabled, and genome size set to 5,000,000 bp (https://github.com/tseemann/shovill). Assembly quality was assessed using QUAST (43) and the EC958 ST131 reference genome GCF_000285655.3.

### Statistical methods.

PCR assay and ST131Typer performances were assessed as both specificity and overall accuracy, in comparison with relevant controls. Reported results included the point estimate and 95% confidence interval.

### Data availability.

ST131Typer is freely available and accessible at https://github.com/JohnsonSingerLab/ST131Typer.
