# Supplementary material for: Novel Multiplex PCR Method and Genome Sequence-Based Analog for High-Resolution Subclonal Assignment and Characterization of Escherichia coli Sequence Type 131 Isolates
Source: Microbiol Spectr. 2022 May 23;10(3):e01064-22. doi: 10.1128/spectrum.01064-22 (PMC9241916; doi:10.1128/spectrum.01064-22)
Supplement: SUPPLEMENTAL FILE 2 — Supplemental material. Download spectrum.01064-22-s002.pdf, PDF file, 0.1 MB [file spectrum.01064-22-s002.pdf]

Supplemental Table 3. *Escherichia coli* isolates (ST131 and non-ST131) used for blinded, independent validation of the novel 3-pool multiplex PCR assay.

Code label[a]	Isolate	ST (phylogroup)	ST131 PCR profile number[b,c]	Subclone[d] (*fimH* allele or O:H type) / clade[d]
Ec-01	H17	ST131 (B2)	4	*H*22 subclone (*fimH27*) / clade B0
Ec-02	JJ1897	ST131 (B2)	5	*H*22 subclone / clade B0
Ec-03	JJ1969	ST131 (B2)	6[e]	*H*22 subclone / clade B1
Ec-04	CD306	ST131 (B2)	9	*H*30S subclone / clade C0[e]
Ec-05	JJ2193	ST131 (B2)	11	*H*30R1 subclone / clade C1
Ec-06	JJ1886	ST131 (B2)	13	*H*30Rx subclone / clade C2
Ec-07	U004	ST131 (B2)	14	*H*30Rx subclone (*fimH35*) / clade C2
Ec-08	U024	ST131 (B2)	12	*H*30R1 subclone / clade C1-M27
Ec-09	U4	ST144 (B2)	n.a.[f]	n.a.[f]
Ec-10	BS488	ST131 (B2)	2	*H*41 subclone (O25b:H4) / clade A
Ec-11	M670745	ST131 (B2)	3	*H*41 subclone (O25b:H5) / clade A
Ec-12	MVAST20	ST131 (B2)	1	*H*41 subclone (O16:H5) / clade A
Ec-13	CD400	ST131 (B2)	6[e]	*H*22 subclone / clade B1
Ec-14	2H16	ST10 (A)	n.a.[f]	n.a.[f]
Ec-15	V27	ST73 (B2)	n.a.[f]	n.a.[f]
Ec-16	2H25	ST95 (B2)	n.a.[f]	n.a.[f]
Ec-17	USVAST306	ST1193 (B2)	n.a.[f]	n.a.[f]
Ec-18	V31	ST12 (B2)	n.a.[f]	n.a.[f]
Ec-19	V10	ST69 (D)	n.a.[f]	n.a.[f]

[a]Ec numbers served as code labels during blinded validation testing in a separate laboratory. Actual strain identities were revealed to the separate laboratory after their PCR assay results had been recorded in the interactive Excel file and validated by the initial laboratory.

[b]The ST131 isolates represented 11 of 15 defined PCR assay profiles (Figure 1,Table 5).

[c]Unique ST131 PCR assay profiles not included in this isolate set: profiles 7 (clade B1, *fimH30*), 8 (clade B1, *fimH*-negative), 10 (clade C0, fluoroquinolone-resistant), and 15 (clade C2, O-nontypable, *H*30Rx).

[d]Subclone labels are per Price et al., clade labels per Petty et al. and Matsumura et al.

[e]ST131 profile 6 was represented by two isolates (JJ1969 and CD400).

[f]n.a., not applicable (non-ST131).
